# B cell receptor signaling drives APOBEC3 expression via direct enhancer regulation in chronic lymphocytic leukemia B cells

**DOI:** 10.1038/s41408-022-00690-w

**Published:** 2022-07-01

**Authors:** Zhiquan Wang, Huihuang Yan, Justin C. Boysen, Charla R. Secreto, Renee C. Tschumper, Dania Ali, Qianqian Guo, Jian Zhong, Jiaqi Zhou, Haiyun Gan, Chuanhe Yu, Diane F. Jelinek, Susan L. Slager, Sameer A. Parikh, Esteban Braggio, Neil E. Kay

**Affiliations:** 1 Division of Hematology, Department of Medicine, Mayo Clinic, Rochester, MN 55905 USA; 2 Division of Computational Biology, Mayo Clinic, Rochester, MN 55905 USA; 3 Department of Immunology, Mayo Clinic, Rochester, MN 55905 USA; 4 Division of Gastroenterology and Hepatology, Mayo Clinic, Rochester, MN 55905 USA; 5 Epigenomics Development Laboratory, Epigenomics Program, Center for Individualized Medicine, Mayo Clinic, Rochester, MN 55905 USA; 6 Shenzhen Institute of Synthetic Biology, Shenzhen Institutes of Advanced Technology, Chinese Academy of Sciences, Shenzhen, 518055 China; 7 The Hormel Institute, University of Minnesota, Austin, MN 55912 USA; 8 Department of Immunology, Mayo Clinic, Scottsdale, AZ 85259 USA; 9 Division of Hematology/Oncology, Department of Medicine, Mayo Clinic, Scottsdale, AZ 85259 USA

**Keywords:** Acute lymphocytic leukaemia, Cancer epigenetics

## Abstract

Constitutively activated B cell receptor (BCR) signaling is a primary biological feature of chronic lymphocytic leukemia (CLL). The biological events controlled by BCR signaling in CLL are not fully understood and need investigation. Here, by analysis of the chromatin states and gene expression profiles of CLL B cells from patients before and after Bruton’s tyrosine kinase inhibitor (BTKi) ibrutinib treatment, we show that BTKi treatment leads to a decreased expression of APOBEC3 family genes by regulating the activity of their enhancers. BTKi treatment reduces enrichment of enhancer marks (H3K4me1 and H3K27ac) and chromatin accessibility at putative APOBEC3 enhancers. CRISPR-Cas9 directed deletion or inhibition of the putative APOBEC3 enhancers leads to reduced APOBEC3 expression. We further find that transcription factor NFATc1 couples BCR signaling with the APOBEC3 enhancer activity to control APOBEC3 expression. We also find that enhancer-regulated APOBEC3 expression contributes to replication stress in malignant B cells. In total we demonstrate a novel mechanism for BTKi suppression of APOBEC3 expression via direct enhancer regulation in an NFATc1-dependent manner, implicating BCR signaling as a potential regulator of leukemic genomic instability.

## Introduction

CLL, the most common leukemia in the U.S. with ~21,000 new cases diagnosed each year, is characterized by a constitutively activated BCR signaling pathway [1]. BCR signaling has a crucial role in both normal B cell development and B cell malignancies. During normal development, B cells are derived from bone marrow hematopoietic stem cells and mature through the expression of a functional BCR. In CLL, the BCR signaling pathway is activated by antigens in the tissue microenvironment or mutations in the BCR signaling genes to promote leukemic cell maintenance and expansion [1, 2]. BCR signaling is mediated through the activation of downstream kinases, such as spleen tyrosine kinase (SYK), Bruton tyrosine kinase (BTK), and phosphoinositide 3-kinases (PI3K) [3]. These kinases have become key therapeutic targets to inhibit BCR signaling in the treatment of B cell malignancies. Indeed, BTK inhibitors (BTKis) such as ibrutinib, acalabrutinib, and zanubrutinib have remarkable efficacy in CLL [4–6].

Cell signaling pathways can regulate gene expression through modification of the epigenetic states [7–9]. Recently, epigenomic studies in CLL detected alterations of epigenetic landscapes as well as mutations of genes encoding key chromatin machinery [10–12], however, the mechanisms and functional importance of these epigenetic programs in BCR signaling are largely unknown. One major mechanism that epigenetic programs utilize to control gene expression and cell states is regulating the activity of enhancers, a class of regulatory DNA elements capable of stimulating transcription over long genomic distances [13]. At enhancers, transcription factors (TFs) trigger the recruitment of chromatin-modifying enzymes to establish active histone modifications on adjacent nucleosomes, such as histone H3 lysine 27 acetylation (H3K27ac) and histone H3 lysine 4 mono-methylation (H3K4me1). As a result, the active enhancers can promote their target gene’s expression and subsequent related cellular functions.

Apolipoprotein B editing complex (APOBEC3) family members are cytidine deaminases that play important roles in responses to retroviruses infections [14]. Recently, APOBEC3 induction has been shown to increase DNA replication stress and chromosome instability in breast and lung cancer evolution [15], and there are also reports showing that APOBEC family mutational signatures are associated with the poor prognosis of multiple myeloma [16]. However, the function of APOBEC3 in CLL is largely unknown.

We reasoned that, exploring the mechanism(s) in CLL B cells whereby BTKi regulates downstream gene expression would provide insights into the understanding of the pathobiological features of the BCR signaling pathway as well as new directions for CLL treatment. Here we report that the BCR signaling pathway drives APOBEC3 expression via NFATc1-dependent enhancer regulation. We also showed that the APOBEC3 enhancer is involved in the process of DNA replication stress, implicating its role in CLL B cell genomic instability.

## Methods

Antibodies, methods for cell culture, plasmids, western blot, and RT-PCR are described in the Supplementary Methods.

### CUT&Tag

CUT&Tag was performed as described (https://www.protocols.io/view/bench-top-cut-amp-tag-bcuhiwt6/abstract). CUT&Tag libraries were sequenced to 50 base pairs on an Illumina HiSeq 4000 using pair-end mode at the Mayo Clinic Gene Analysis Shared Resources.

### ATAC-seq

ATAC-seq library construction was performed as previously described [17, 18]. Fifty thousand cells were lysed in cold ATAC-Resuspension Buffer (RSB) (0.1% NP40, 0.1% Tween 20, and 0.01% digitonin) and then washed out with cold ATAC-RSB (0.1% Tween 20) followed by centrifugation at 4 °C. Pellets were resuspended in a transposition mix containing Tagment DNA buffer, Tn5 Transposase, and 0.05% Tween 20 and incubated for 30 min at 37 °C. Transposed DNA was purified and then amplified using Nextera sequencing primers (Illumina) and NEB High Fidelity 2X PCR Master Mix. PCR-amplified DNA was purified and sequenced using an Illumina HiSeq 4000 with paired-end reads of 50 bases.

### CRISPR-mediated enhancer deletion

sgRNAs targeting both sides of the individual enhancers with designed and cloned to lentiGuide-Puro (Addgene plasmid # 52963). MEC1 cell with stable Cas9 expression with infected with the paired sgRNAs targeting the enhancer and selected by puromycin for 1 week. The deletion of the enhancers was validated by PCR with primers across the whole enhancer region.

### CRISPR-dCas9-Krab-MECP2 inactivation

gRNAs targeting AEs were cloned to lentiGuide-Puro. MEC1 cells were infected with pLX-TRE-dCas9-KRAB-MeCP2-BSD and selected with 10 µg/ml Blasticidin, then the pool populations were infected with two gRNAs targeting each enhancer region and selected with Puromycin. Two days after selection and the cells were incubated with doxycycline (0.5 µg/ml) for 5 days then harvested for analysis.

### Statistical analysis

Significance was defined as *p* ≤ 0.05 unless otherwise noted in the text (*p < 0.05; **p < 0.01; ***p < 0.001). Paired or unpaired two-tailed Student’s *t*-tests and two-way ANOVA statistics were calculated using GraphPad Prism 9. Two-tailed Student’s *t*-tests were used for pairwise comparisons as noted in the text.

## Results

### BCR signaling regulates APOBEC3 expression in CLL

**Fig. 1:**
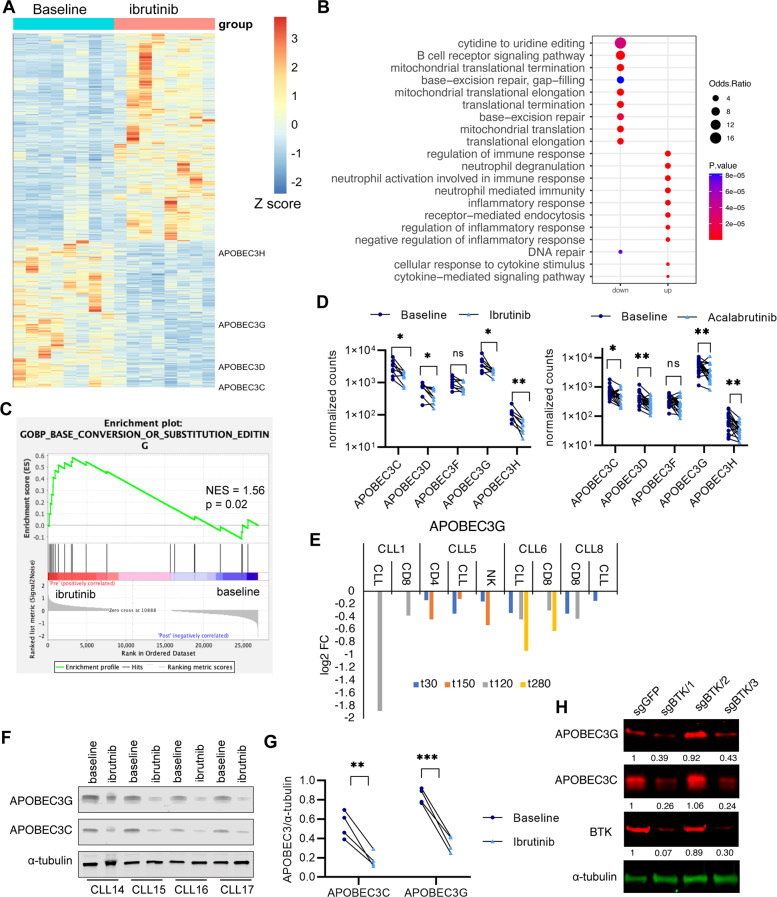
BCR signaling-dependent APOBEC3 expression in CLL B cells. **A** Heatmap representing the expression of significant changes in genes (= 3334, up = 1964, down = 1370. *p* < 0.05, fold change >1.5) (in CLL B cells from the same CLL patients before and with one-year continuous ibrutinib treatment. The gene expression was determined by RNA-seq, *n* = 8 patients. **B** Gene Ontology (GO) enrichment analysis for the ibrutinib suppressed genes. CLL B cells were harvested from the same CLL patients before and with one-year continuous ibrutinib treatment and the gene expression was determined by RNA-seq, *n* = 8 patients. **C** Gene set enrichment analysis showing enrichment of base conversion or substitution editing linked genes in ibrutinib pretreated CLL B cells compared to CLL B cells from patients with continuous 1-year ibrutinib treatment. **D** APOBEC3 gene expression changes in CLL B cells from ibrutinib-treated patients compared to that of pretreated patients. baseline = pre ibrutinib treatment; ibrutinib = 1-year of continuous ibrutinib treatment. **E** APOBEC3G expression in indicated cells from ibrutinib-treated patients. t30 means 30 days of ibrutinib treatment. **F** Immunoblot analysis of APOBEC3C and APOBEC3G in CLL B cells from patients before and after 1-year ibrutinib treatment. baseline = pre ibrutinib treatment; ibrutinib = 1-year ibrutinib treatment. **G** Quantification of the western blot intensity in panel (**F**). **H** Immunoblot analysis of APOBEC3C and APOBEC3G in MEC1 cells infected with indicated BTK sgRNAs, the intensity of each blot analysis was quantified and normalized against α-tubulin, with the normalized intensity of each blot in sgGFP control cells set to 1. The whole-cell lysates were harvested 5 days after infection.

To explore BCR signaling-regulated genes in CLL, we analyzed the gene expression profile of CLL B cells from eight patients before, and after 1-year of continuous ibrutinib treatment by mRNA-seq. Detailed patient information is included in Supplementary Tables 1, 2 and their precise IGHV status is included in Supplementary Table 3. BTKi treatment-induced dramatic gene expression changes (total changed genes = 3334, up = 1964, down = 1370, *p* < 0.05, fold change >1.5) (Fig. 1A, Supplementary Fig. 1A, and Supplementary Table 4). Interestingly, compared to the downregulated genes, upregulated genes with ibrutinib treatment are more patient-specific, thus we focused our subsequent analysis on the downregulated genes. As reported before, ibrutinib treatment suppresses the expression of genes involved in mitochondrial function [19] and the BCR signaling pathway (Fig. 1B, Supplementary Tables 5, 6, and Supplementary Fig. 1B). In addition to these pathways known to be involved in B cell malignancies and CLL survival, we found that ibrutinib treatment led to the reduction of expression in genes associated with single-strand DNA deamination [20] (Fig. 1B, C and Supplementary Fig. 1C). The BTKi regulated DNA deamination genes mainly contain the APOBEC3 family genes (*APOBEC3C, APOBEC3D, APOBEC3F, APOBEC3G,* and *APOBEC3H*) [21], and their expression levels showed a consistent reduction in CLL B cells from ibrutinib-treated patients (Fig. 1D). Analysis of the published dataset (**Published dataset A**, Supplementary Table 7) [22] showed that APOBEC3 expression was also suppressed by another BTKi, acalabrutinib, in leukemic cells of CLL patients (Fig. 1D). Consistent with the previous report [23], *AICDA* (AID) expression was also downregulated by BTKi treatment (Supplementary Table 4). We further analyzed published single-cell RNA-seq data (**Published dataset B**, Supplementary Table 7) [12] and found that BTKi treatment downregulated the expression of APOBEC3C in CLL B cells from two patients and APOBEC3G in CLL B cells of all four patients (Fig. 1E and Supplementary Fig. 1D, E), which indicates that the BTKi induced downregulation of APOBEC3s is generally consistent among most of the CLL B cells. We confirmed the reduction of APOBEC3 levels by RT-qPCR and western blot in CLL B cells from patients before and after 1-year of continuous ibrutinib treatment (Fig. 1F, G and Supplementary Fig. 1F–H). It is possible that the reduced expression of APOBEC3 genes by ibrutinib treatment is due to the elimination of cells with high expression of these genes, thus we treated the purified primary CLL B cells in vitro with ibrutinib at a sublethal level and still found reduced APOBEC3 expression (Supplementary Fig. 1I, J), indicating the reduced expression of these genes is not due to the elimination of APOBEC3 high expressed cells. We also showed that depletion of BTK in the BCR signaling-dependent MEC1 and JEKO1 cells decreased APOBEC3C and APOBEC3G levels (Fig. 1H and Supplementary Fig. 1K), which further confirmed that BCR signaling regulates APOBEC3 expression. To further determine the role of BTKi treatment on APOBEC3 expression, we evaluated the APOBEC3 expression of CLL B cells from patients at sequential stages of ibrutinib treatment (Baseline, on ibrutinib treatment, and then at their relapse, *n* = 4 patients, Supplementary Tables 1–3) by RNA-seq. Our results showed that ibrutinib treatment-induced decreased APOBEC3 gene expression, but the APOBEC3 expression started to return to the baseline level (pre-therapy) with continued treatment except for patient CLL11 (Supplementary Fig. 2). In relapse, the APOBEC3 expression level was restored to levels comparable to that of the baseline samples in two patients (CLL10 and CLL12) (Supplementary Fig. 2). These results suggest that the reduction of APOBEC3 is associated with effective ibrutinib treatment.

We also compared the basal expression of APOBEC3 between CLL B cells and normal B cells using published RNA-seq datasets (**Published dataset C and D**, Supplementary Table 7) [11, 24] and found an increased expression of APOBEC3 in CLL B cells compared to the normal B cells (Supplementary Fig. 3A, B). We then confirmed the increased expression of APOBEC3C and APOBEC3G by western blot in CLL B cells compared to normal B cells (Supplementary Fig. 3C, D). Together, these results indicate that active BCR signaling is required for APOBEC3 expression in leukemic cells of CLL patients.

To test if direct BCR activation leads to upregulation of APOBEC3 genes in CLL cells, we treated the primary CLL cells derived from four different CLL patients with CpG or IgM and measured the expression of the APOBEC3 genes by RT-PCR. However, we did not see consistent upregulation of APOBEC3s with these treatments (Supplementary Fig. 3E); it is possible that the upregulation of APOBEC3s by BCR signaling may require long-term chronic treatment.

### BTKi treatment suppresses the activity of putative enhancers of APOBEC3

**Fig. 2:**
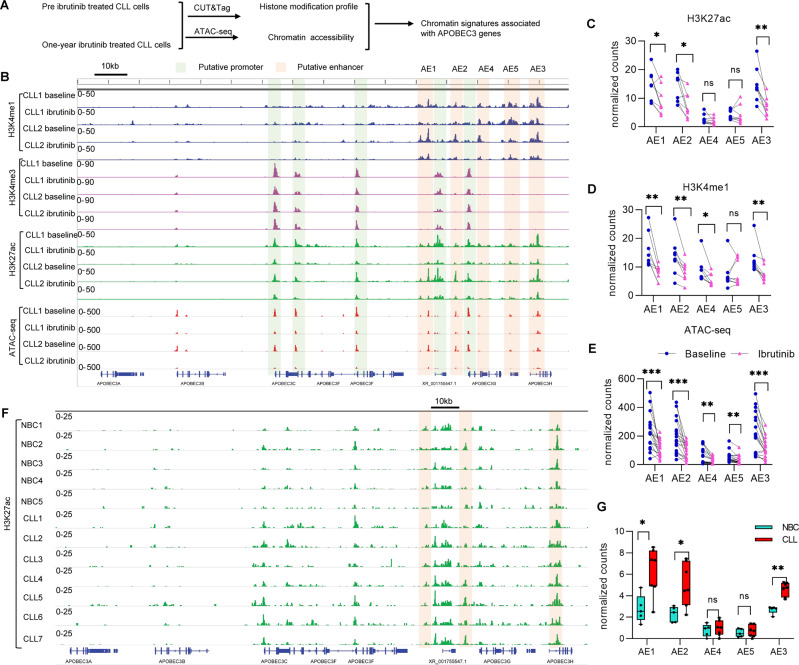
Ibrutinib treatment suppresses the activity of putative enhancers of APOBEC3. **A** Schematic view of the analysis of epigenetic signatures of CLL B cells with 1-year of continuous ibrutinib treatment. **B** Genome tracks showing CUT&Tag of H3K4me1, H3K4me3, H3K27ac, and ATAC-seq profiles of putative APOBEC3 enhancers. Light green and brown shadows show promoters and enhancers respectively. baseline = pre ibrutinib treatment; ibrutinib = 1-year of continuous ibrutinib treatment. **C**–**E** Normalized read counts of the CUT&Tag (*n* = 8) and ATAC-seq (*n* = 18) profiles in panel (**B**). **F** Genome tracks showing H3K27ac profiles of APOBEC3 enhancers from CUT&Tag on normal and CLL B cells. The CLL and normal B cells were purified by negative selection of unwanted cells with tetrameric antibody complexes recognizing non-B cells and glycophorin A on red blood cells (RBCs) from PBMCs. **G** Normalized read counts of H3K27ac CUT&Tag profile in panel (**F**) were shown (NBC, *n* = 5; CLL, *n* = 7).

Signaling pathways can regulate chromatin-modifying enzymes, histone modifications, and nucleosome occupancy to affect both the epigenetic and transcriptional state of cells [7, 8]. We hypothesized that BCR signaling regulates APOBEC3 expression by modifying the local chromatin states around the APOBEC3 gene cluster. To check the local chromatin states of APOBEC3, we performed CUT&Tag [25] and ATAC-seq to map the histone marks including H3K4me1, H3K4me3, H3K27ac, and examined the chromatin accessibility of the leukemic cells from CLL patients before and with one-year of continuous ibrutinib treatment (Fig. 2A and Supplementary Tables 1, 2). We found the enrichment of enhancer mark H3K4me1 proximal to the APOBEC3 gene clusters, and these regions were also enriched with the active enhancer mark H3K27ac and had an open chromatin state (Fig. 2B and Supplementary Fig. 4). This suggests that this region is a putative enhancer(s) that controls APOBEC3 expression. Hereafter we refer to this region as APOBEC3 enhancers (AEs). We further found that BTKi treatment caused reductions of H3K4me1, H3K27ac, and chromatin accessibility at these regions in most ibrutinib-treated patients, however, there was no change in the promoter marker H3K4me3 of these genes (Fig. 2B–E and Supplementary Fig. 4), which indicated that BTKi treatment leads to APOBEC3 gene expression changes via the regulation of their enhancer activity. Next, we analyzed the published ATAC-seq data of CLL B cells from patients who are on ibrutinib treatment (**Published dataset E**, Supplementary Table 7) [12]. While the ibrutinib treatment duration in this study was shorter than our study (30–120 days after treatment), we noted it still led to modest reductions of chromatin accessibility at AEs in most patients (Supplementary Fig. 5A, B). We also analyzed the H3K27ac profile from another published work (**Published dataset F**, Supplementary Table 7) [26], and found that ibrutinib treatment could also decrease the H3K27ac at the AEs in this study (Supplementary Fig. C, D). We next checked the chromatin accessibility around APOBEC3 gene clusters in CLL B cells from the four patients at different stages of ibrutinib treatment (Baseline, on ibrutinib treatment, and then at relapse, Supplementary Tables 1, 2) by ATAC-seq. Consistent with the RNA-seq results (Supplementary Fig. 2), chromatin accessibility at AEs was suppressed by ibrutinib treatment at the early time point and was returning to pre-therapy levels with continued treatment, and at relapse was restored to the level comparable to that of the baseline samples in three patients (Supplementary Fig. 6A, B). For patient CLL11, while ibrutinib treatment did not change APOBEC3 expression as dramatically as in the other three patients, we did not observe the chromatin accessibility change either in this patient (Supplementary Figs. 2, 6). Thus, these results indicate that effective BTKi therapy can induce decreased expression of APOBEC3 genes through regulation of the activity of their enhancers.

Given the above findings, we further analyzed the published datasets (**Published dataset C**, Supplementary Table 7) [11] to compare the enhancer signatures between CLL B cells and normal B cells. CLL B cells showed higher enrichment of H3K27ac and increased chromatin accessibility at AEs when compared to normal B cells (Supplementary Fig. 7A). Then we performed the H3K27ac CUT&Tag in normal B cells and CLL B cells and our results also found that CLL B cells had higher H3K27ac levels in the AE regions (Fig. 2F, G). Analyzing the ENCODE H3K27ac ChIP-seq data (**Published dataset G**, Supplementary Table 7) we found that this specific enhancer signature is limited to the B cell lineages in hematopoietic cell populations (Supplementary Fig. 7B). Importantly, the Hi-C [27] data generated from B-lymphocyte cell line GM12878 cells [28] showed that the APOBEC3 genes and enhancers had high levels of genomic interactions and are located in the same topologically associating domain (TAD) (Supplementary Fig. 7C). We did not see the change of expression of other genes located in the same TAD by BTKi treatment (Supplementary Table 8), which suggests the regulation of gene expression by AEs is limited to the APOBEC3 genes. Together, our results indicate that the expression of APOBEC3 may be controlled by BCR signaling through enhancer regulation.

### APOBEC3 expression is controlled by their enhancer activity

**Fig. 3:**
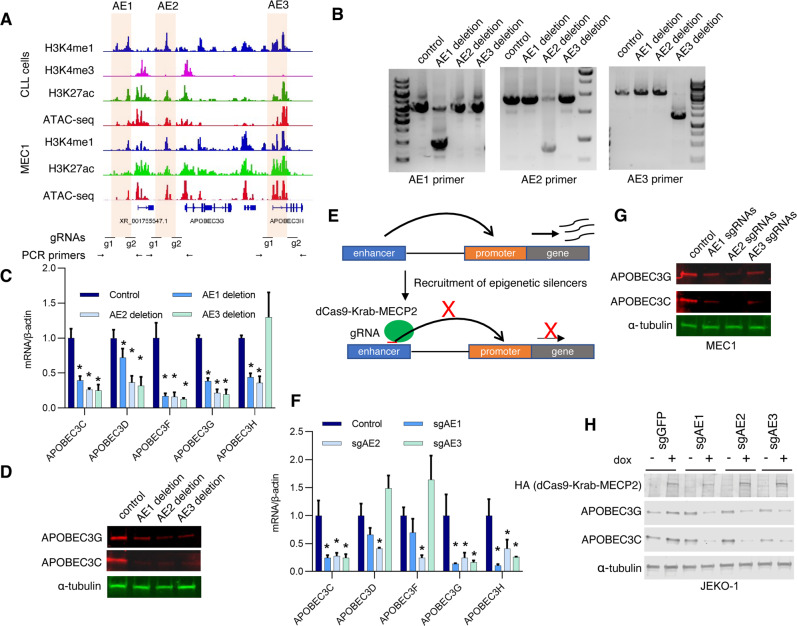
Deletion or inhibition of APOBEC3 enhancers suppresses APOBEC3 expression. **A** Outline of the strategy to delete the AEs by CRISPR-Cas9. The genomic locus of the gRNAs to target the AEs and the PCR primers to amplify the enhancer regions were shown. **B** Gel imaging shows the successful deletion of AEs. The AE regions were amplified by PCR primers spanning the indicated regions as in panel (**A**). **C** RT-qPCR analysis of APOBEC3 expression in the AE-deleted MEC1 cells. *n* = 3 independent experiments. **D** Western blot analysis of APOBEC3C and APOBEC3G expression in the AEs deleted MEC1 cells. **E** Schematic view of the strategy of CRISPRi. MEC1 cells expressing dCas9-Krab-MECP2 were infected with sgRNAs targeting the indicated enhancer regions to suppress the enhancer activity. **F** RT-qPCR analysis of APOBEC3 expression after inhibition of individual AEs by CRISPRi. *n* = 3 independent experiments. The cells with infection of indicated sgRNAs were incubated with 0.25 μg/ml doxycycline for 3 days to induce dCas9-Krab-MECP2 expression before the cells were harvested. **G** Western blot analysis of APOBEC3C and APOBEC3G expression after inhibition of individual AEs by CRISPRi in MEC1 cells. The cells with infection of indicated sgRNAs were incubated with 0.25 μg/ml doxycycline for 3 days before the cells were harvested. **H** Western blot analysis of APOBEC3C and APOBEC3G expression after inhibition of individual AEs by CRISPRi in JEKO1 cells. The JEKO1 cells with dCas9-Krab-MECP2 infected with indicated sgRNAs were incubated with or without 0.25 μg/ml doxycycline for 3 days before the cells were harvested. In the AEs deletion and CRISPRi assays, gRNA targeting GFP is used as a control.

Based on the enrichment of H3K4me1, H3K27ac, and chromatin accessibility, AE regions contain five active enhancer modules, and we have designated these modules as AE1, AE2, AE3, AE4, and AE5 (Fig. 2B). Because the base level of H3K27ac enrichment of AE4 and AE5 is relatively low and not decreased significantly by ibrutinib treatment, we subsequently focused our study on the AE1, AE2, and AE3 (Figs. 2B, 3A). To assess the functional activity of these enhancers on the expression of APOBEC3 genes, we investigated the consequence of the deletion of each one of these AEs in the MEC1 cell line. MEC1 was used based on fact that MEC1 has been utilized in a previous CLL epigenetic study [29]. We found that MEC1 has similar chromatin states at the AEs, though the relative enrichment of enhancer marks (H3K4me1, H3K27ac, and ATAC-seq intensity) at AE1 and AE2 is lower compared to that of the primary CLL B cells (Fig. 3A). Initially, we tried to generate AE knockout clones by CRISPR-mediated knockout, however, we failed to grow cells from single clones with sgRNA transfected MEC1 cells (data not shown). Next, we infected the Cas9 expressing MEC1 cells with sgRNAs to generate pooled populations of cells with AEs deletion (gRNA locations and PCR strategy are shown in Fig. 3A). PCR analysis confirmed the deletion of AE1, AE2, and AE3 (Fig. 3B). Both deletions of AE1 or AE2 reduced the expression of APOBEC3 genes (Fig. 3C, D), while AE3 deletion suppressed the expression of APOBEC3C, APOBEC3D, APOBEC3F, and APOBEC3G, but not APOBEC3H (Fig. 3C, D).

To exclude that the observed results with AE deletions above were outcomes of undesired changes induced by Cas9 [30–32], we used CRISPR interference (CRISPRi) to modulate enhancer activity by rewriting the epigenetic states without changing the underlying DNA sequence [31, 32]. In the CRISPRi assay, a KRAB effector domain and a MECP2 fused to a catalytically dead Cas9 (dCas9-Krab-MECP2) are recruited to the enhancers by CRIPSR gRNAs, where these epigenetic suppressors generate a suppressive chromatin state [31, 32]; in this manner, the expression of the target gene of the enhancer will be inhibited (Fig. 3E). RT-PCR and western blot analysis showed a significant reduction of most of the APOBEC3 genes and protein expression by gRNAs targeting these three AEs (Fig. 3F, G). We further performed CRISPRi experiments in JEKO1, another B cell malignancy line, which also has active APOBEC3 enhancers (Supplementary Fig. 8F) and found that inhibition of these enhancers could suppress APOBEC3C and APOBEC3G expression (Fig. 3H).

We also tested the expression of other neighborhood genes (SUN2, CBX6, CBX7, Supplementary Table. 8) of AEs and found that AEs depletion or inhibition did not change their expression (Supplementary Fig. 7D–G). Together, we identified the BCR signaling-dependent enhancers that regulate APOBEC3 expression. All three enhancer modules (AE1, AE2, and AE3) can regulate most of the APOBEC3 gene expression. Thus, these results are consistent with the recent report that individual elements of a super-enhancer region could contribute to their target gene expression [33].

### NFATc1 controls APOBEC3 enhancer activity

**Fig. 4:**
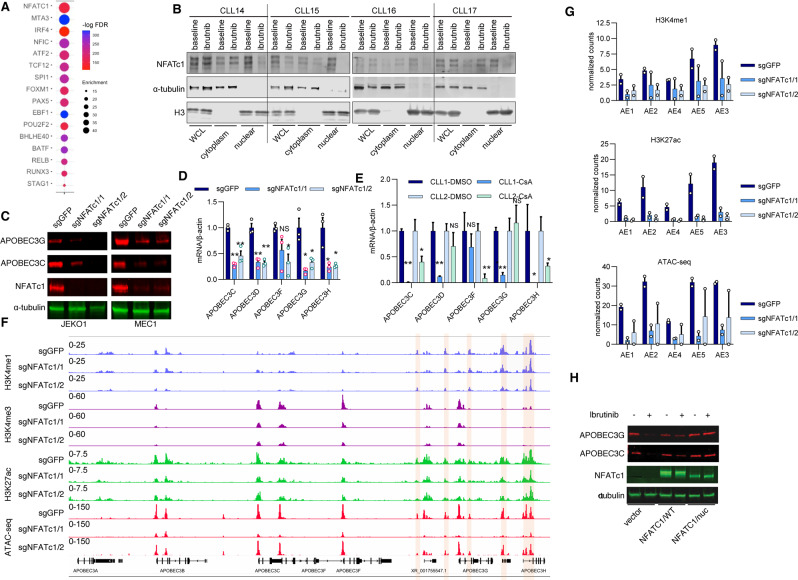
NFATc1 controls the APOBEC3 expression through enhancer regulation. **A** Top TF motifs enriched in the regions with decreased chromatin accessibility in CLL B cells after 1-year of continuous ibrutinib treatment are shown. The differential ATAC-seq regions between ibrutinib and baseline CLL samples were analyzed by *tfmotifviews* [61] and randomly matched control regions were generated as control. **B** Western blot showing protein levels of NFATc1 in nuclear and cytoplasm fraction of CLL B cells from patients treated with or without ibrutinib. The samples were collected as in Fig. 1A. **C** The APOBEC3C and APOBEC3G levels were analyzed by western blot in NFATc1 depleted MEC1 and JEKO1 cells. Indicated cells were infected with sgRNAs targeting NFATc1 for 5 days and the whole-cell lysates were harvested for western blot. The intensity of each blot analysis was quantified and normalized against α-tubulin, with the normalized intensity of each blot in sgGFP control cells set to 1. **D** RT-qPCR analysis of APOBEC3 expression in NFATc1 depleted MEC1 cells. *n* = 3 independent experiments. Indicated cells were infected with sgRNAs targeting NFATc1 for 5 days before the RNA was purified and analyzed by RT-PCR. **E** RT-qPCR analysis of APOBEC3 expression in CLL B cells treated with 2.5 μM Cyclosporin A for 24 h. **F** Genome tracks showing CUT&Tag of H3K4me1, H3K4me3, H3K27ac, and ATAC-seq profiles of APOBEC3 genes in NFATc1 depleted and control MEC1 cells. The cells were treated as in panel (**D**). **G** Normalized read counts of the CUT&Tag and ATAC-seq results in panel (**F**), *n* = 2 independent experiments for each histone mark. **H** Western blot showing protein levels as indicated in wildtype (NFATc1/wt) or nuclear stable form (NFATc1/nuc) NFATc1 expressing MEC1 cells treated with or without ibrutinib at 2.5 µM for 3 days. NFAT1c1 was detected by Flag antibody.

It has been shown that pioneer transcription factors can recruit other transcription factors, nucleosome remodeling complexes, and histone modifiers to reprogram chromatins, thereby initiating the formation of an activating or repressive regulatory sequence [34]. Therefore, we reasoned that identifying transcription factors (TF) enriched at regions with altered chromatin states would allow us to determine the mechanism that links BCR signaling to epigenetic regulation of AE activity and APOBEC3 expression in CLL B cells. To that end, we evaluated whether specific TF motifs were enriched within regions with chromatin accessibility reduction after ibrutinib treatment. The top enriched motifs included binding sites for the NFATc1, ATF2, and IRF4 (Fig. 4A). We focused on NFATc1 (also called NFAT2 [35]), a TF that locates in both promoters and enhancers [36] and can promote enhancer reprogramming [37]. Importantly, NFATc1 is a putative downstream factor of the BCR in CLL [38, 39] and has been shown to regulate APOBEC3G expression [38, 39]. We observed a reduction of nuclear NFATc1 level in CLL B cells of ibrutinib-treated patients compared to that of the ibrutinib pretreated patients (Fig. 4B and Supplementary Fig. 8A). We further found that treating the primary CLL B cells with ibrutinib for 24 h in vitro also resulted in the depletion of nuclear NFATc1 (Supplementary Fig. 8B). These results suggest that BTKi treatment depletes the nuclear fraction of NFATc1, which may in turn abolish its function. We found that BTKi treatment led to a reduction of NFATc1 mRNA levels (Supplementary Fig. 8C) and protein levels in some of the CLL samples (Fig. 4B and Supplementary 8A). Although NFATc2 (also called NFAT1 [35]) binds to similar DNA motifs as NFATc1, we did not see changes in NFATc2 with ibrutinib treatment (Supplementary Fig. 8C). These results suggested the role of NFATc1 in the regulation of AE activity and APOBEC3 family gene expression in leukemic B cells.

We next explored the function of NFATc1 in the regulation of APOBEC3 expression. Depletion of NFATc1 led to a reduced expression of APOBEC3s in MEC1 and JEKO1 cell lines (Fig. 4C, D), whereas depletion of another top hit TF IRF4 (Fig. 4A) had no effect on APOBEC3 expression (Supplementary Fig. 8D, E). Importantly, treating the primary CLL B cells with cyclosporin A (CsA), a calcineurin inhibitor known to suppress the NFATc1 nuclear localization [40], suppressed APOBEC3 expression (Fig. 4E). We next performed CUT&Tag and ATAC-seq in NFATc1 depleted and control MEC1 cells to test if NFATc1 is required for the APOBEC3 enhancer activity. NFATc1 depletion resulted in decreased chromatin accessibility and H3K27ac enrichment at AEs (Fig. 4F, G and Supplementary Fig. 8F, G), which demonstrated that NFATc1 is the key factor that maintains the active chromatin state of APOBEC3 enhancers.

To further test our hypothesis that BTKi treatment depletes nuclear NFATc1 to abolish the AEs activity and APOBEC3 expression, we generated a MEC1 cell line expressing a nuclear stable form of NFATc1 (NFATc1^nuc^), which is unable to be phosphorylated and constitutively located in the nucleus [41]. We found that ibrutinib treatment suppressed APOBEC3 expression in NFATc1 wildtype but not NFATc1^nuc^ expressing MEC1 cells (Fig. 4H). This result strongly supports that nuclear depletion of NFATc1 is required for the BTKi-induced APOBEC3 expression reduction.

### APOBEC3 enhancers contribute to DNA replication stress in CLL B cells

**Fig. 5:**
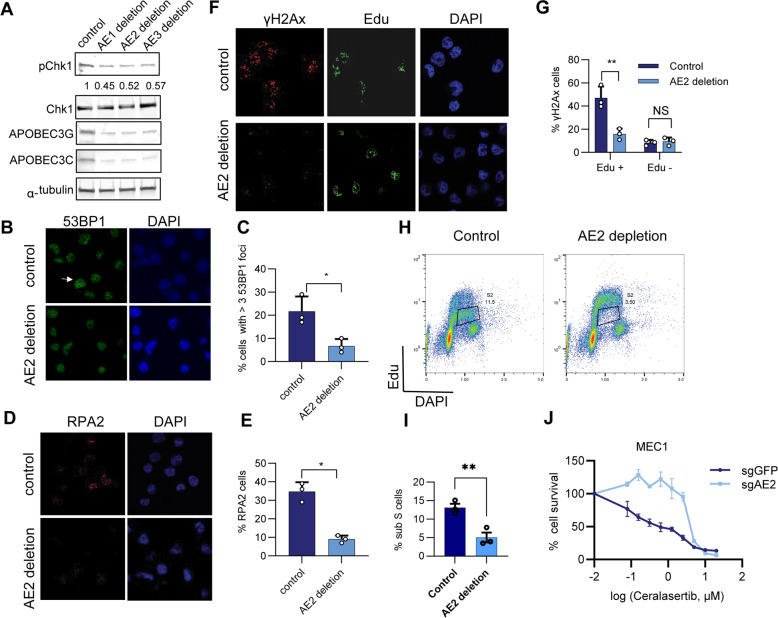
APOBEC3 enhancers contribute to the replication of stress-induced DNA damage in MEC1 cells. **A** Western blot analysis of pChk1 level in the indicated AE-deleted MEC1 cells. **B** Representative images of 53BP1 staining in AE2 depleted and control MEC1 cells. The intensity of pChk1 was quantified and normalized against total Chk1, with the normalized intensity of control cells set to 1. **C** The quantification of cells with more than three 53BP1 foci in panel (**B**). One hundred cells were counted per experiment, *n* = 3 independent experiments. **D** Representative images of RPA staining in AE2 depleted and control MEC1 cells. **E** The quantification of cells with RPA2 in panel (**D**). One hundred cells were counted per experiment, *n* = 3 independent experiments. **F** Representative images of γH2AX and Edu staining of AE2 depleted and control MEC1 cells treated. Cells were incubated with 10 µM Edu for 30 min before harvest. **G** The quantification of S phase cells with γH2AX in panel (**F**), 100 cells were counted per experiment, *n* = 3 independent experiments. **H** AE2 was deleted and control MEC1 cells were incubated with Edu for 30 min before the cells were stained with Click-it Alexa 488 azide and DAPI. **I** The quantification of sub-S phase cells percentage in (**H**) **J** AE2 deleted and control MEC1 cells were incubated with ATR inhibitor Ceralasertib as indicated for 5 days, and the cell viability was determined by MTS.

Next, we sought to determine the role of APOBEC3 enhancers in CLL B cells in relation to DNA stress. We evaluated the function of APOBEC3 in MEC1 cells by deletion of the APOBEC3 enhancers or only AE2, which could downregulate the expression of most of the APOBEC3 genes (Figs. 3C, D, 5A). We found that MEC1 cells have a high level of spontaneous DNA damage, illustrated by phosphorylated pChk1 (S345) [42], 53BP1 nuclear body (a marker of DNA replication stress [43]), accumulation of RPA2 positive cells [44], and DNA damage marker gamma H2Ax (γH2Ax) in the S phase cells [45] (Fig. 5A–G). However, AE-deleted MEC1 cells showed a reduction of pChk1 (S345) compared to MEC1 control cells (Fig. 5A). In addition, compared to the control cells, AE2 deleted cells had fewer 53BP1 nuclear bodies (Fig. 5B, C) and RPA2 positive cells (Fig. D, E), both of which are associated with replication stress-induced DNA damage response. Importantly, AE2 deleted cells also showed decreased γH2Ax in the S phase cells (Fig. F, G). To check the role of APOBEC3 enhancer on DNA replication, we performed Edu/PI assay in AE2 deleted cells. Consistently, we noticed that MEC1 cells had a fraction of S phase cells with low Edu incorporation during the S phase, indicating DNA replication stress [46] (Fig. 5H, I); however, AE2 deletion greatly increased Edu incorporation (Fig. 5H, I). Because cancer cells with a high level of replication stress are particularly sensitive to ATR inhibition, we then treated the control and AE2 deletion MEC1 cells with ATR inhibitor Ceralasertib [47]. We found that while the control MEC1 cells were sensitive to Ceralasertib, AE2 depletion suppressed the sensitivity of MEC1 cells to Ceralasertib (Fig. 5J). Taken together, these data suggest that increased expression of APOBEC3 may be involved in DNA replication stress and have the potential to drive genomic instability in malignant B cells.

## Discussion

In recent years, the implementation of highly specific, targeted novel agents for human malignancies has greatly improved the outcome of certain diseases. We have reasoned that patient-derived tissues while on or after novel agent targeted therapy can provide powerful tools to study the epigenetic and gene expression regulatory networks related to the targeted pathway. Supporting this concept, we have explored the epigenome and transcriptome of CLL B cells from patients before, during, and after the ibrutinib treatment and demonstrated that the BCR signaling pathway in leukemic B cells regulates APOBEC3 genes expression via direct regulation of their enhancers. Overall, using ibrutinib-treated CLL patients as a model, we demonstrate that this novel targeted therapy agents can be used to gain important insights and provides a valuable resource for the study of basic biologic questions.

The IGHV mutation status impacts the affinity of the BCR for antigen and the extent of BCR signaling activity, which implicates a potential role of IGHV status in the BCR signaling-regulated APOBEC3 expression. However, with the limited sample numbers of our current cohort, it is difficult to conclude that there is any correlation between IGHV mutation status and BTKi-induced APOBEC3 reduction. Despite the interindividual heterogeneity of APOBEC3 expression, we found a consistent reduction of APOBEC3 levels with effective ibrutinib treatment. Consistently, although the base level of AE activities is heterogeneous, the reduction of chromatin accessibility and active histone marks at AEs with ibrutinib treatment is consistent amongst the patient samples we tested. Thus, we conclude that reduced APOBEC3 expression in response to the BTKi treatment results from inhibition of BCR signaling and their subsequent modification of enhancer activity.

Signaling pathways can regulate gene expression through modification of the epigenetic states of the cells [7, 8], and most of this knowledge has been gained from stem cell studies [48, 49]. This mechanism is also utilized by oncogenic signaling pathways to activate the transcription of genes that promote malignant cell survival and growth [17, 50, 51]. Our results provide evidence that a signaling pathway which regulates gene expression through epigenetic modification exists in leukemic CLL B cells as well. As previously shown [52], we found that NFATc1 is activated by the BCR signaling pathway in CLL cells. The active NFATc1 in turn activates the enhancer activity of the APOBEC3 genes to promote their expression. Although NFATc1 has been shown to be involved in the regulation of Epstein Barr virus (EBV) associated super-enhancers, the exact mechanism by which NFATc1 controls the activity of enhancers in CLL is unknown. Previous studies have shown that NFATc1 binds to several chromatin regulators (e.g., p300) [53], so it is possible that NFATc1 works to recruit the chromatin modification enzymes and remodelers to these regions to generate an open chromatin structure for APOBEC3 expression. Indeed, our data show that NFATc1 depletion leads to decreased chromatin accessibility and active histone modifications at the AEs. Future studies exploring the chromatin regulators that work with NFATc1 to regulate the chromatin states should provide more information about this regulatory aspect in the mechanism of APOBEC3 expression.

BCR signaling can activate PI3K and pharmacological targeting of PI3K (e.g., idelalisib) is also a therapeutic strategy in chronic lymphocytic leukemia [54, 55], though current use of PI3Ki in CLL is diminishing due to undue toxicities and lesser efficacy. It has been known that PI3K/Akt can activate NFATc1 activity in multiple systems [52, 56], which indicated that PI3K may mediate the interaction between BCR and NFATc1. Therefore, it would be interesting to see if suppressing BCR signaling by PI3K inhibitors like idelalisib also downregulates APOBEC3 expression, which would provide more information to elucidate the mechanisms whereby BCR regulates downstream epigenetic events.

Modification of APOBEC3 expression by BTKi treatment may be associated with clinical benefits but there are also potential clinical complications. APOBEC3 family members play important roles in intrinsic responses to infection by retroviruses and have been implicated in the control of other viruses, such as parvoviruses, herpesviruses, papillomaviruses, hepatitis B virus, and retrotransposons [20]. There are reported cases showing the reactivation of the hepatitis B virus (HBV) after ibrutinib treatment [57, 58]. Because APOBEC3G can also inhibit HBV, our finding may provide new insights to the understanding of HBV reactivations with ibrutinib treatment where imbalances or deficiencies of APOBEC3 family members contribute to deficient host response to infections.

APOBEC3 genes are implicated in the generation of genomic mutations of various types of cancers [21], thus we speculate that they can also drive gene mutations during the evolution of CLL. If true, our findings here would provide mechanistic insights that the BCR signaling pathway regulated enhancer remodeling couples the extracellular environment in the regulation of the genetic evolution of leukemic cells. Indeed, our preliminary analysis shows that APOBEC3 genes are involved in DNA replication stress in malignant B cells. We also found that enhancer-regulated APOBEC3 expression is associated with DNA damage during the S phase in the MEC1 cell line, which suggests that increased expression of APOBEC3 may induce transcription replication conflicts, a major driver of cancer evolution [59].

It has been reported that loss of NFATc1 results in the acceleration of clonal evolution in CLL and Richter’s transformation [60], while our results in contrast support that NFATc1-controlled APOBEC3 expression may promote clonal evolution. This inconsistency can be explained by two possibilities: (1) NFATc1 target genes are involved in multiple cell functions, while APOBEC3 promotes DNA replication stress, other target genes may suppress this process; (2) Suppression of CLL evolution by NFATc1 and the biological role of NFATc1-controlled APOBEC3 may function at different stages of CLL progression.

Future work will focus on the role of the BCR-regulated APOBEC3 expression in relation to alteration of immune resistance as it relates to infection propensity as well as the role of the APOBEC3 gene family in B cell genomic instability and CLL clonal evolution.

## Supplementary information

SUPPLEMENTAL data and methods

supplementary table 1

supplementary table 2

Supplementary table 3

supplementary table 4

supplementary table 5

supplementary table 6

supplementary table 7

supplementary table 8

supplementary table 9

## Data Availability

The datasets generated during and/or analysed during the current study are available from the corresponding author (wang.zhiquan@mayo.edu) on reasonable request.
