# Supplementary material for: B cell receptor signaling drives APOBEC3 expression via direct enhancer regulation in chronic lymphocytic leukemia B cells
Source: Blood Cancer J. 2022 Jul 1;12(7):99. doi: 10.1038/s41408-022-00690-w (PMC9249768; doi:10.1038/s41408-022-00690-w)
Supplement: Supplementary file 1 — SUPPLEMENTAL data and methods [file 41408_2022_690_MOESM1_ESM.pdf]

B Cell Receptor Signaling Drives APOBEC3 Expression Via Direct Enhancer Regulation in Chronic Lymphocytic Leukemia B Cells

Zhiquan Wang[1,10], Huihuang Yan[2], Justin C. Boysen[1], Charla R. Secreto[1], Renee C Tschumper[3], Dania Ali[1], Qianqian Guo[4], Jian Zhong[5], Jiaqi Zhou[6], Haiyun Gan[6], Chuanhe Yu[7], Diane F Jelinek[8], Susan L. Slager[1,2], Sameer A. Parikh[1], Esteban Braggio[9], Neil E. Kay[1,10]

Affiliations:

[1] Division of Hematology, Department of Medicine, Mayo Clinic, Rochester, MN 55905 USA.

[2] Division of Computational Biology, Mayo Clinic, Rochester, MN 55905 USA.

[3] Department of Immunology, Mayo Clinic, Rochester, MN 55905, USA.

[4] Division of Gastroenterology and Hepatology, Mayo Clinic, Rochester, MN 55905, USA

[5] Epigenomics Development Laboratory, Epigenomics Program, Center for Individualized Medicine, Mayo Clinic, Rochester, MN 55905, USA.

[6] Shenzhen Institute of Synthetic Biology, Shenzhen Institutes of Advanced Technology, Chinese Academy of Sciences, Shenzhen 518055, China.

[7] The Hormel Institute, University of Minnesota, Austin, MN 55912, USA.

[8] Department of Immunology, Mayo Clinic, Scottsdale, AZ 85259, USA.

[9] Division of Hematology/Oncology, Department of Medicine, Mayo Clinic, Scottsdale, AZ 85259, USA.

[10] Correspondence: wang.zhiquan@mayo.edu, kay.neil@mayo.edu,

This file includes:

Supplementary Materials and Methods

Supplementary Figures 1 to 8

References for Supplementary Materials and Methods

Materials and methods

Antibodies	Vendor	Cat Num and Antibody ID	Assays
Mouse anti-NFATc1 (7A6)	Santa Cruz Biotechnology	Cat# sc-7294, RRID: AB_2152503	Western blot
NFATc1 (D15F1) Rabbit mAb	Cell Signaling Technology	Cat# 8032, RRID: AB_10829466	Western blot
Mouse anti-α-tubulin (IB)	Developmental Studies Hybridoma Bank	Cat#: 12G10, RRID: AB_1157911	Western blot
Rabbit polyclonal to Histone H3	Abcam	Cat#: 12 ab1791, RRID: AB_302613	Western blot
Rabbit polyclonal to H3K4me1	Abcam	Cat# ab8895, RRID: AB_306847	CUT&Tag
Rabbit polyclonal to H3K4me3	Abcam	Cat# ab8580, RRID: AB_306649	CUT&Tag
Rabbit polyclonal to H3K27ac	Abcam	Cat#: ab4729, RRID: AB_2118291	CUT&Tag
Mouse monoclonal ANTI-FLAG® M2	Sigma-Aldrich	Cat#: F1804, RRID: AB_262044	Western blot
APOBEC3G (D9C6Z) Rabbit mAb	Cell Signaling Technology	Cat# 43584, RRID: AB_2799245	Western blot
Rabbit polyclonal to APOBEC3C	GeneTex	Cat# GTX102164, RRID: AB_2616091	Western blot
Rabbit polyclonal to 53BP1	Novus	Cat# NB100-304, RRID: AB_10003037	immunofluorescence
Anti-phospho-Histone H2A.X (Ser139) Antibody	Millipore	Cat# 05-636, RRID: AB_309864	immunofluorescence
Phospho-Chk1 (Ser345) (133D3) Rabbit mAb antibody	Cell Signaling Technology	Cat# 2348, RRID: AB_331212	Western blot
Chk1 (2G1D5) Mouse mAb antibody	Cell Signaling Technology	Cat# 2360, RRID: AB_2080320	Western blot
Anti-RPA 32 kDa subunit Antibody (9H8)	Santa Cruz Biotechnology	Cat# sc-56770, RRID: AB_785534)	immunofluorescence
RDye® 800CW donkey anti-mouse IgG	LI-COR Biosciences	Cat#: 926-32212, RRID: AB_621847	Western blot
IRDye® 680RD donkey anti-rabbit IgG	LI-COR Biosciences	Cat#: 926-68073 RRID: AB_10954442	Western blot

Goat Anti-Mouse IgG (H+L) Antibody, Alexa Fluor 594 Conjugated	Thermo Fisher Scientific	Cat# A-11005, RRID: AB_141372	immunofluorescence
Goat Anti-Rabbit IgG (H+L) Antibody, Alexa Fluor 488 Conjugated	Thermo Fisher Scientific	Cat# A-11008, RRID: AB_143165	immunofluorescence

Published datasets. The information of the datasets retrieved from online database is included in **Supplementary Table 7**.

DNA oligonucleotides. The sequences of the all the oligos used in this study are included in **Supplementary Table 9.**

Cell culture. All cell lines were maintained at 37°C in a humidified incubator with an atmosphere of 5% CO_2. HEK293T/c17 (HEK293T) cells were cultured in Dulbecco's Modified Eagle's Medium (DMEM; Corning, Corning, NY); MEC1 and JEKO-1 cells were cultured in RPMI-1640 medium (Corning). Media were supplemented with 10% fetal bovine serum (FBS; Invitrogen), 2 mM L-glutamate (Corning), and 1x penicillin/streptomycin solution (Invitrogen). MEC1 and JEKO-1 cells stably expressing Cas9 or dCas9-KRAB-MECP2 were cultured in MEC1 medium supplemented with 10µg/ml Blasticidin.

Purification of leukemic and normal B cells. All patients providing research blood samples provided written informed consent according to the Declaration of Helsinki to the Mayo Clinic Institutional Review Board, which approved these studies. Informed consents were also obtained from healthy donors to obtain their blood B cells. The patients' relevant information is shown in **Supplementary Table 2** and **3**. Primary CLL B cells and normal B cells were purified from blood samples using the RosetteSep B cell enrichment kit (Stem Cell Technologies). The typical purification range of CD5+/CD19+ CLL B cells was > 95% as determined by flow cytometric analysis. The assays utilized with these CLL B cells samples were included in **Supplementary Table 1**. CLL B cells were cultured for optimum viability in serum-free AIM-V (Gibco) medium as previously described[1]. Normal B cells were purified from blood samples using the RosetteSep B cell enrichment kit (Stem Cell Technologies) with CD19+ CLL B cells where the purity was > 95%.

IGHV analysis. Total RNA was isolated by the TRIzol method (Life Technologies, Grand Island, NY) and 2 µg of RNA was converted to cDNA using the Bio-Rad iScript Select cDNA Synthesis Kit (Bio-Rad, Hercules, CA). To determine the IGHV gene usage, 2 µl of cDNA is amplified using the Qiagen HotStarTaq MasterMix kit (Qiagen, Valencia, CA) in 7 individual PCR

reactions (one for each IGHV family) using 0.5 μM of sense primer in conjunction with 0.5 μM of antisense primer to the IgM constant region. An additional reaction for beta actin is included for each cDNA.

Amplification was carried out in a Perkin Elmer 9700 thermocycler (Perkin Elmer, Waltham, MA, USA) using the following conditions: 95 ^{0}C for 15 min; 35 cycles of 95 ^{0}C for 30 s, 60 ^{0}C for 60 s, 72 ^{0}C for 60 s and a final cycle of 72 ^{0}C for 10 min. Amplified products were visualized on a 1.5% agarose TAE gel with ethidium bromide, excised and purified with the Wizard SV Gel and PCR Clean-Up Kit (Promega, Madison, WI). Products are sequenced directly by Sanger Sequencing using the IgM primer used for amplification. Resulting sequences are aligned to germline IGHV region gene sequences using ImMunoGeneTics Information (IMGT) System reference sets and IMGT/VQuest software (http://imgt.cines.fr). Subset analysis is done by ARResT/Assign http://tools.bat.infspire.org/arrest/assignsubsets/.

RNA-seq. Total RNA was extracted using the Direct-zol RNA Kit (Zymo Research). Library preparation and sequencing were performed using the NovaSeq 6000 platform, paired-end 150 bp by Novogene (Sacramento, CA).

RNA-seq data analysis. Reads were aligned using Kallisto[2]. Transcript abundance files were then used in the DESeq2 R package, which was used for all downstream differential expression analysis and generation of volcano plots. Differentially expressed genes between samples from baseline and ibrutinib treatment patients were compared with a cutoff of fold Change > 1.5, $p<0.05$.

CUT&Tag data analysis. CUT&Tag data were analyzed following CUT&Tag Data Processing and Analysis Tutorial (https://www.protocols.io/view/cut-amp-tag-data-processing-and-analysis-tutorial-bjk2kkye). Reads were aligned to the human genome (hg38) or E. coli genome using Bowtie2 [17]. The fragments mapped to E. coli were used as the Spike-in calibration. Uniquely mapped reads were used for further analysis. Peaks were identified using MACS2 [18] using 0.01 as the cutoff FDR value. Mapped reads were transformed to BigWig files by deepTools. Read density at enhancer regions were calculated by deepTools multiBigwigSummary.

ATAC-seq data analysis. ATAC-seq data analysis was performed as previously reported [21-23]. Nextera adapter sequences were first trimmed from the reads by Trim Galore!. These reads were aligned to the hg38 reference genome using bowtie2 with standard parameters and a maximum fragment length of 2,000. Picard was used to remove duplicate reads. De-duplicated reads were filtered for high quality (MAPQ ≥ 30), nonmitochondrial, non-Y chromosome, and

properly paired (Samtools flag 0 × 2) reads. TF motifs enrichment were analyzed by tfmotifviews and random matched control regions was generated as control[24]. TFmotifView relies on known TF motifs from the curated, non-redundant, vertebrates JASPAR CORE 2020 database[25].

Quantitative RT-PCR (RT-qPCR). Total RNA was extracted using the Direct-zol RNA Kit (Zymo Research, Tustin, CA). Reverse transcription into cDNA was performed using Superscript III and random hexamer primers (Life Technologies) according to the manufacturer's instructions. Quantitative PCR (qPCR) was carried out using iTaq Universal SYBR Green Supermix (Biorad) on a CFX96 Touch Deep Well Real-Time PCR System (Biorad) using primers listed below. Relative expression was calculated as 2^- (ΔCt (target)- ΔCt (GAPDH/β-actin)) and normalized to the indicated controls. Three independent experiments were performed; for each experiment, and gene expression was assessed in triplicate.

Nuclear protein extraction. Pelleted cells were resuspended with buffer 1 (15mM Tris-HCl (pH 7.5), 60 mM KCl, 15 mM NaCl, 5 mM $MgCl_2$, 1 mM $CaCl_2$, 0.25 M Sucrose with 1 mM PMSF, 1 mM DTT, and Complete Protease Inhibitor cocktail tablet added immediately before use) at roughly 5 times the volume of the pellet and gently pipetted up and down to dissociate the pellet. Samples were incubated on ice for 5 minutes and then an equal volume of buffer 1 with 0.4% NP- 40 was added to the sample. Samples were then mixed by inversion for 5 minutes at 4 ^{0}C. Half the of samples were saved and boiled with 1/3 volume of 4xSDS loading buffer as whole cell lysates. The left samples were spun at 200xG for 10 minutes at 4 ^{0}C to pellet nuclei. The supernatant (cytoplasmic fraction) was transferred to a new tube and boiled with 1/3 volume of 4XSDS loading buffer. Pellets were resuspended gently in 0.5mL buffer 1 to wash nuclei, then pelleted again and supernatant was discarded. Nuclear pellet were then boiled with 1X SDS samples buffer before analyzed by western blot.

Western blotting. Whole cell lysates were extracted in Laemmli buffer (60 mM Tris-Cl pH 6.8, 2% SDS, 10% glycerol, 5% β-mercaptoethanol, 0.01% bromophenol blue), separated by SDS-PAGE gel, and transferred to PDVF membrane (MilliporeSigma). Blots were blocked using Odyssey Blocking Buffer (LI-COR Biosciences, Lincoln, NE) prior to incubation with primary antibody at 4°C overnight. Secondary antibody incubations (anti-mouse, 1:5,000 dilution; anti-rabbit, 1:10,000 dilution; both LI-COR Biosciences) were performed for 1 hour at room temperature. Proteins of interest were visualized by Odyssey infrared imaging system (LI-COR Biosciences).

Plasmids. pLX-TRE-dCas9-KRAB-MeCP2-BSD was a gift from Andrea Califano (Addgene plasmid # 140690; http://n2t.net/addgene:140690; RRID: Addgene_140690). lentiGuide-Puro

was a gift from Feng Zhang (Addgene plasmid # 52963; http://n2t.net/addgene:52963; RRID: Addgene_52963). lentiCas9-Blast was a gift from Feng Zhang (Addgene plasmid # 52962; http://n2t.net/addgene:52962; RRID:Addgene_52962).

Lentivirus production. Lentiviral plasmids were co-transfected into HEK293T cells with packaging vectors psPAX2 (Addgene plasmid #12260; a gift from Didier Trono) and pMD2.G (Addgene plasmid #12259; a gift from Didier Trono) using PEI 4000 (Polysciences). Virus-containing medium was collected 48 hours after transfection and cleared of potential cells using 0.45-μm Steriflip filter units (MilliporeSigma).

Nuclear form of NFATc1 expression. Wildtype NFATc1 plasmid pMIG-hNFATc1/bC - AVITEV was a gift from Ria Baumgrass (Addgene plasmid # 74059; http://n2t.net/addgene:74059 ; RRID:Addgene_74059) and a nuclear stable form of NFATc1 (the unphosphorylated) plasmid pUHD.NFATc1nuc was a gift from Jerry Crabtree (Addgene plasmid # 40616 ; http://n2t.net/addgene:40616 ; RRID:Addgene_40616). Both wildtype and nuclear stable NFATc1 cDNA were subcloned to lentiCas9-Blast (Addgene plasmid # 52962; http://n2t.net/addgene:52962; RRID:Addgene_52962) by Infusion cloning (Takara, 638920) to replace Cas9. Lentivirus were packaged in HEK293t cells and then the MEC1 cell line was infected with the virus and selected with 10 μg/ml Blasticidin for stable expression of wildtype and nuclear stable NFATc1.

CRISPR-cas9 knockout. Single guide RNAs (sgRNAs) targeting NFATc1 were designed using the Broad Institute Genetic Perturbation Platform and cloned to lentiCRISPR V2. Lentivirus was packaged, mixed with polybrene (final concentration 8 μg/mL; MilliporeSigma), and transduced to cells. The cells were selected by puromycin before harvested for indicated assays.

Reference

1. Boysen J, Nelson M, Magzoub G, et al. Dynamics of microvesicle generation in B-cell chronic lymphocytic leukemia: implication in disease progression. *Leukemia*. 2017;31(2):350-360.
2. Bray NL, Pimentel H, Melsted P, Pachter L. Near-optimal probabilistic RNA-seq quantification. *Nat Biotechnol*. 2016;34(5):525-527.
3. Rendeiro AF, Krausgruber T, Fortelny N, et al. Chromatin mapping and single-cell immune profiling define the temporal dynamics of ibrutinib response in CLL. *Nat Commun*. 2020;11(1):577.
4. Holmes KB, Sadreev II, Rawstron AC, et al. Ibrutinib induces chromatin reorganisation of chronic lymphocytic leukaemia cells. *Oncogenesis*. 2019;8(5):32.

5. Beekman R, Chapaprieta V, Russiñol N, et al. The reference epigenome and regulatory chromatin landscape of chronic lymphocytic leukemia. *Nature Medicine*. 2018;24(6):868-880.
6. Dunham I, Kundaje A, Aldred SF, et al. An integrated encyclopedia of DNA elements in the human genome. *Nature*. 2012;489(7414):57-74.
7. Wang Y, Song F, Zhang B, et al. The 3D Genome Browser: a web-based browser for visualizing 3D genome organization and long-range chromatin interactions. *Genome Biology*. 2018;19(1):151.

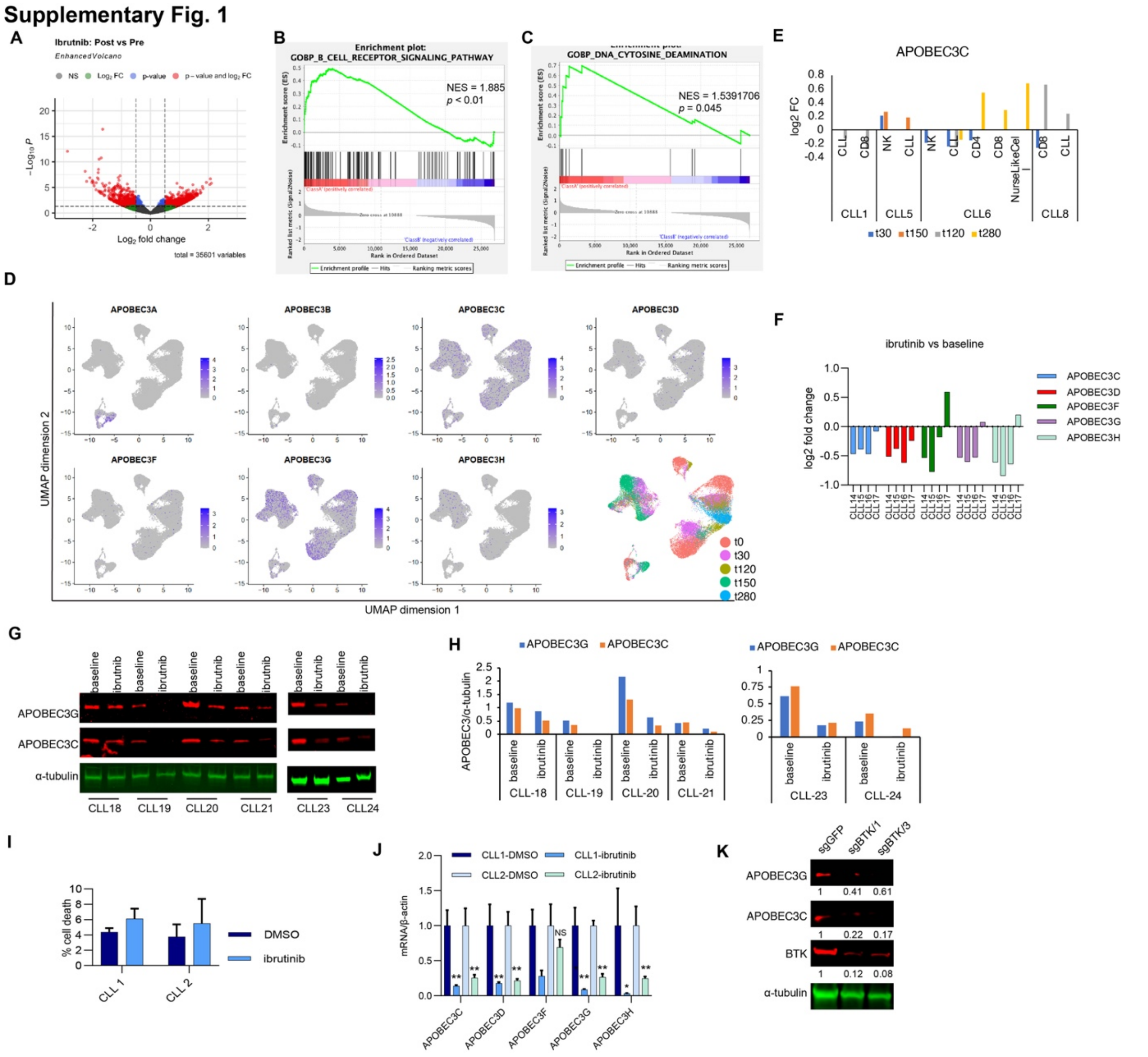

Supplementary Fig. 1 Ibrutinib treatment induces gene expression changes in CLL B cells. (**A**) Volcano blot showing differentially expressed genes in CLL B cells from one-year of continues ibrutinib treated patients compared to that from pretreated patients. (**B**) Gene set enrichment analysis showing enrichment of BCR signaling pathway signatures in CLL cells from patients prior to ibrutinib treatment compared to CLL cells from patients with one-year of

continuous ibrutinib treatment. (**C**) Gene set enrichment analysis showing enrichment of DNA cytosine deamination gene signatures in CLL B cells from patients prior to ibrutinib treatment compared to that from patients after one-year of continuous ibrutinib treatment. The purple color scale represents the normalized counts of defined genes in each cell. (**D**) APOBEC3C expression in indicated cells from ibrutinib treated patients. t30 means 30 days after initiation of ibrutinib treatment. The single cell RNA seq data were obtained from published dataset GSE111015[3]. (**E**) Feature plots showing the expression of APOBEC3C of indicated cell types as in panel (D). (**F**) RT-qPCR analysis of APOBEC3 expression of CLL B cells from patients pre and after one-year ibrutinib treatment. baseline = pre ibrutinib treatment; ibrutinib = one-year of continuous ibrutinib treatment. (**G**) Immunoblot analysis of APOBEC3C and APOBEC3G in CLL B cells from patients before and after one-year ibrutinib treatment. baseline = pre ibrutinib treatment; ibrutinib = one-year ibrutinib treatment. (**H**) Quantification of the western blot intensity in panel (G). (**I**) the cell viability of CLL cells treated with 2.5μM ibrutinib for 3 days was determined by Annexin V/PI staining. (J) RT-qPCR analysis of APOBEC3 in CLL B cells treated in vitro with 2.5μM ibrutinib for 3 days. n = 3 independent experiments for the CLL B cells from each patient. (**K**) Immunoblot analysis of APOBEC3C and APOBEC3G in JEKO1 cells infected with indicated BTK sgRNAs. The whole cell lysates were harvested 5 days after infection.

Supplementary Fig.2

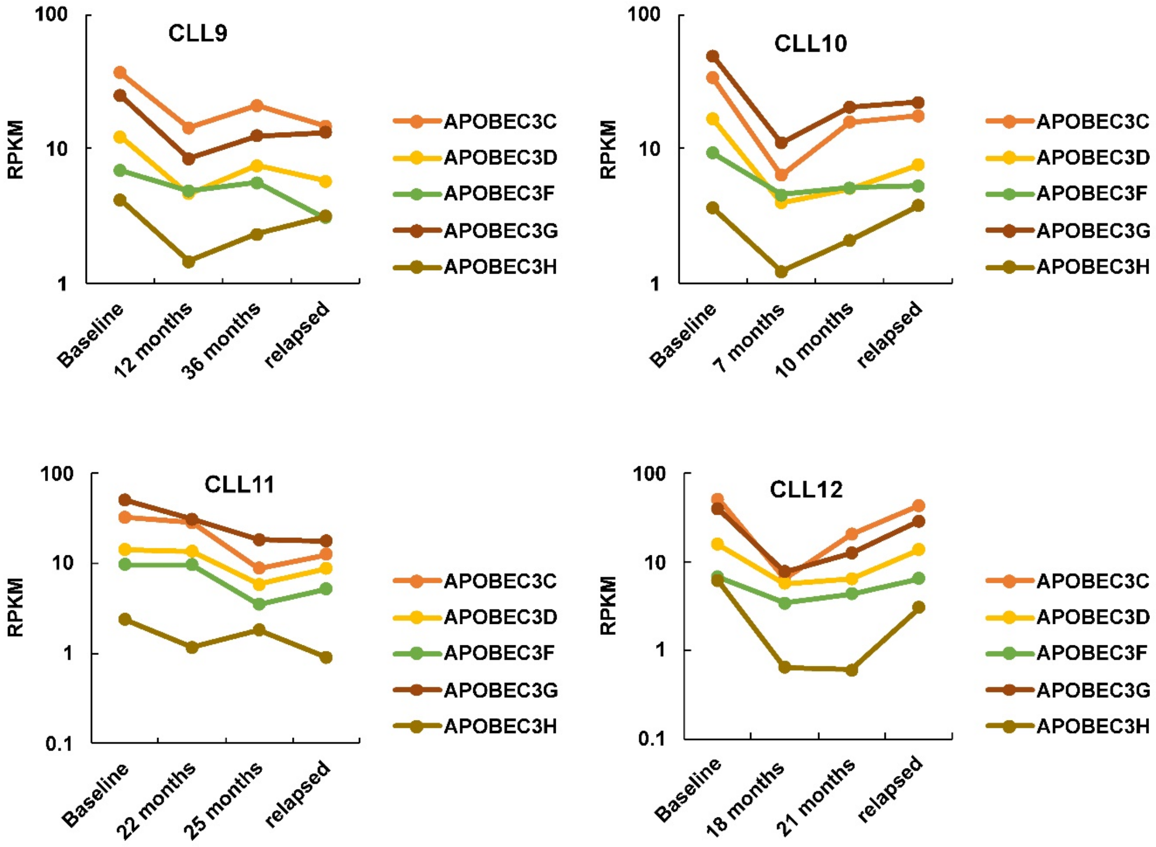

Supplementary Fig. 2 APOBEC3 gene expression changes in CLL B cells from ibrutinib treated patients where we compare the gene expression over time for 4 CLL patients. Gene expression level was determined by RNA-seq.

Supplementary Fig. 3

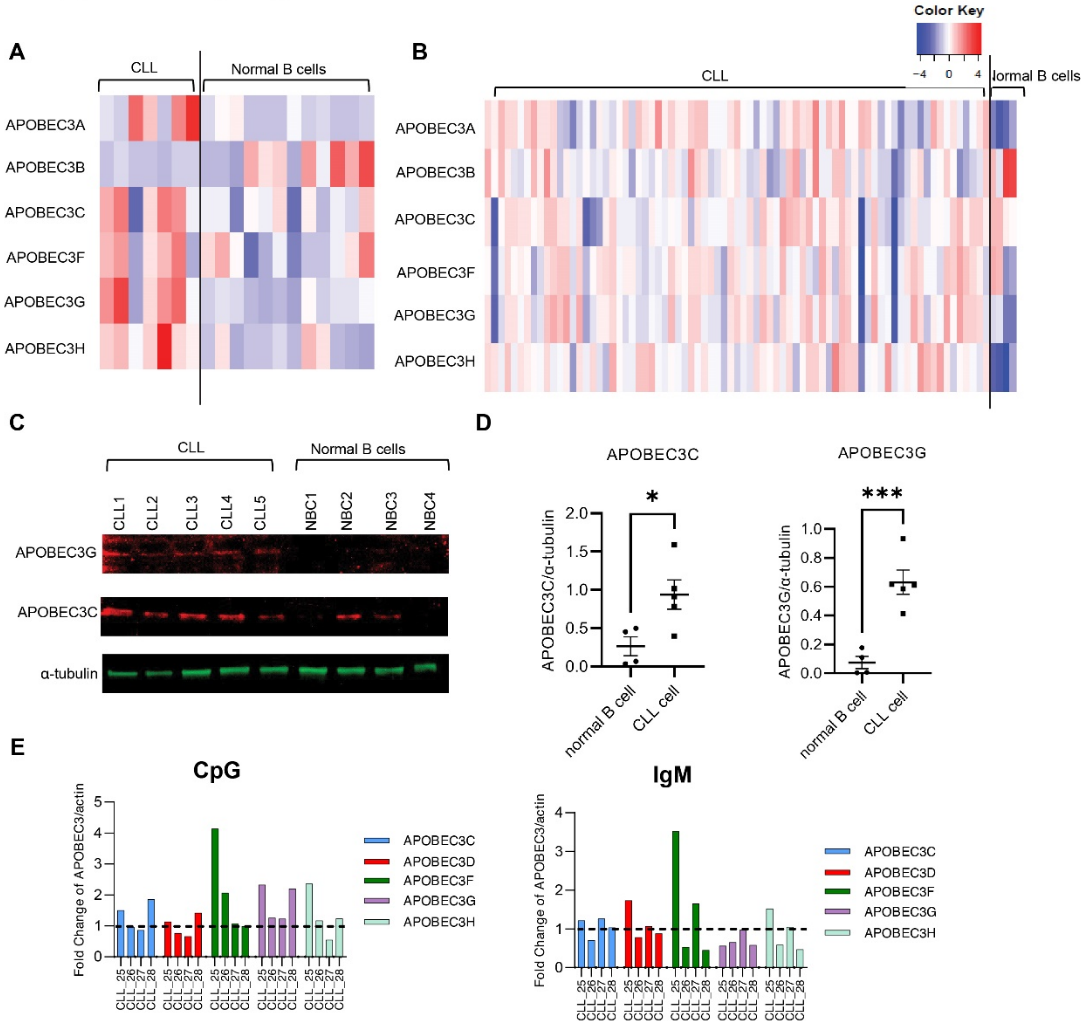

Supplementary Fig. 3 APOBEC3 expression in normal and CLL B cells. (**A** and **B**), Heatmaps showing the relative expression of APOBEC3s in normal B cells and CLL B cells. RNA-seq datasets were obtained from EGAD00001004046 (A) and GSE119103 (B). (**C**) Western blot showing APOBEC3C and APOBEC3G levels in CLL B cells and normal B cells. (**D**) quantification of the western blot intensity is shown in panel (C). (**E**), RT-qPCR analysis of APOBEC3 in CLL B cells treated in vitro with CpG ODN (2.5 µg/ml) or IgM (10 µg/ml) for 2 days.

Supplementary Fig. 4

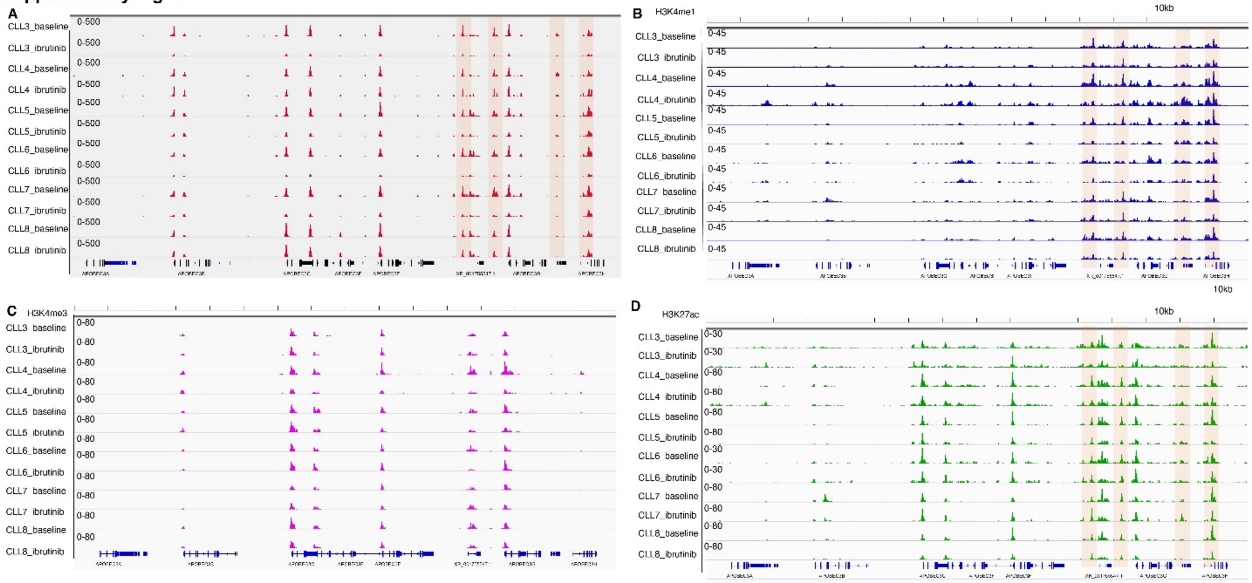

Supplementary Fig. 4 Ibrutinib treatment leads to decreased chromatin accessibility, H3K4me1 and H3K27ac enrichment at APOBEC3 enhancers in CLL B cells. (**A-D**) Genome tracks showing ATAC-seq, CUT&Tag of H3K4me1, H3K4me3, and H3K27ac profiles of putative APOBEC3 enhancers. Light brown shadows show enhancers. baseline = pre ibrutinib treatment; ibrutinib = one-year of continuous ibrutinib treatment.

Supplementary Fig. 5

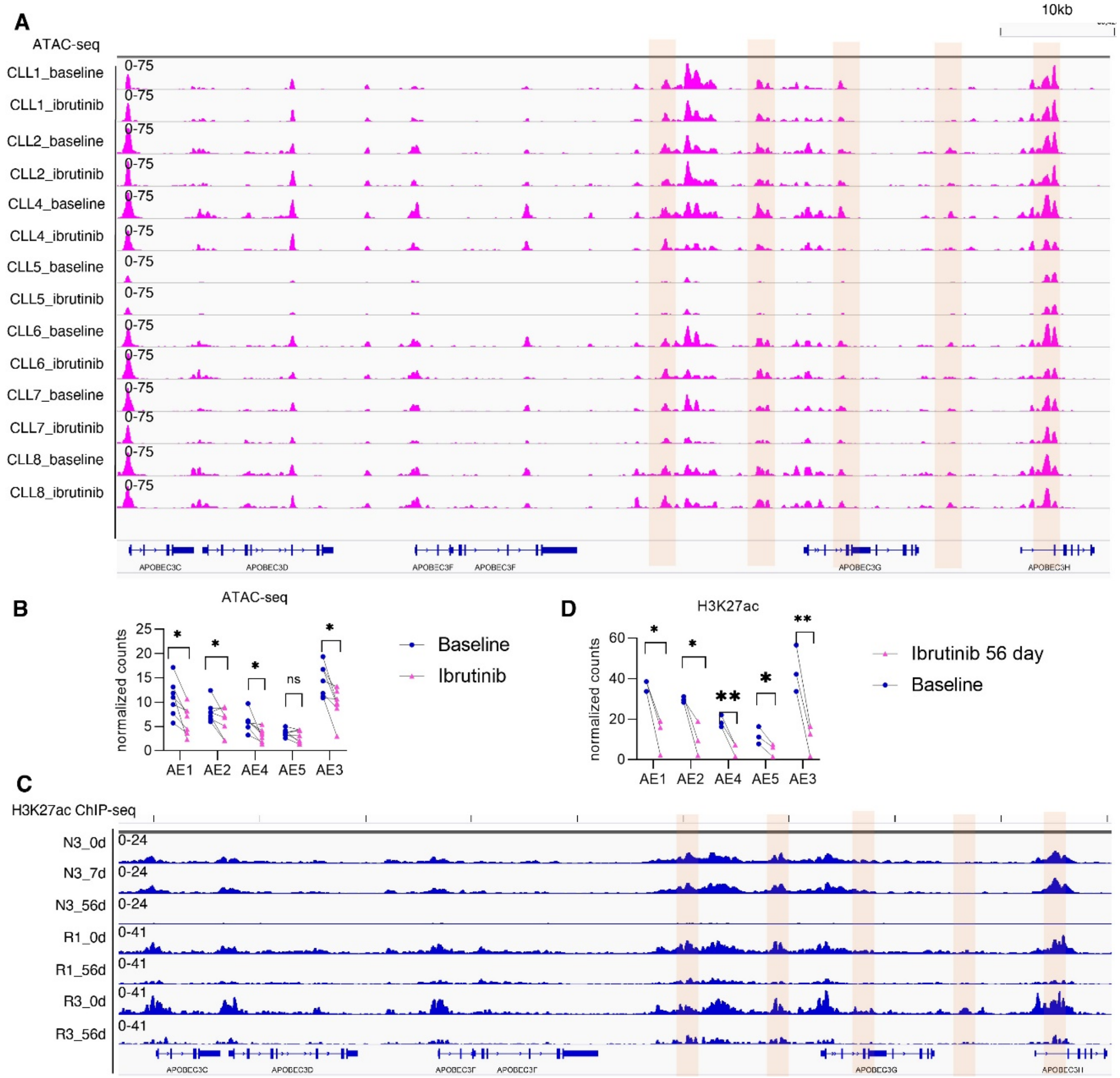

Supplementary Fig. 5 Ibrutinib treatment leads to decreased chromatin accessibility at APOBEC3 enhancers. (**A**), Genome tracks showing ATAC-seq profiles of putative APOBEC3 enhancers. baseline = pre ibrutinib treatment; ibrutinib = 120 days post initiation of ibrutinib treatment. Data are downloaded from GSE111015. (**B**) Normalized read counts of ATAC-seq at the putative APOBEC3 enhancers of panel A. (**C**), Genome tracks showing H3K27ac ChIP-seq profiles of putative APOBEC3 enhancers. Data are downloaded from AssayExpress E-MTAB-6410[4]. (**D**) Normalized read counts of ChIP-seq at the putative APOBEC3 enhancers of panel C.

Supplementary Fig. 6

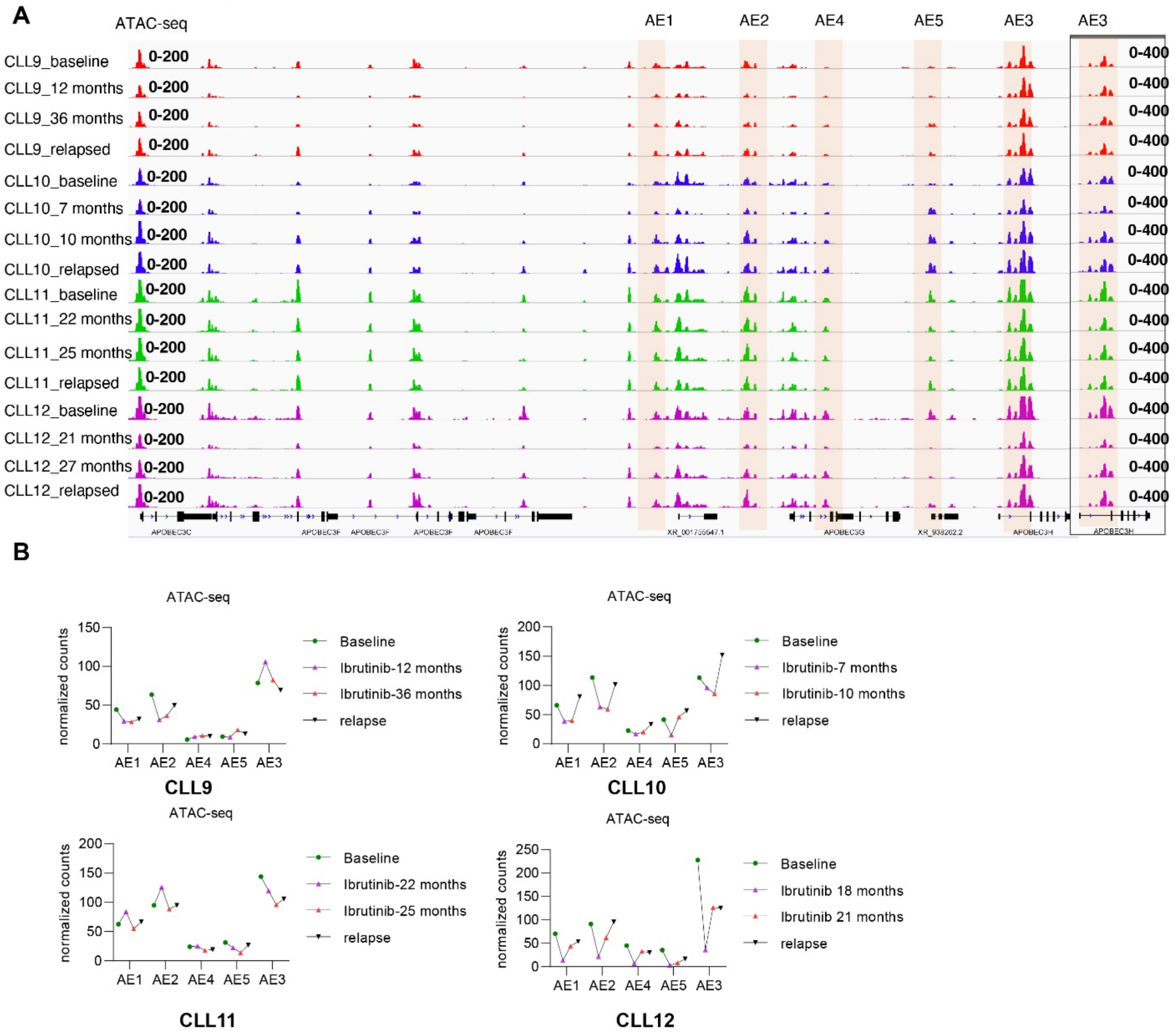

Supplementary Fig. 6 Effective ibrutinib treatment leads to decreased chromatin accessibility at APOBEC3 enhancers. (**A**) Genome tracks showing ATAC-seq profiles of putative APOBEC3 enhancers. baseline = pre ibrutinib treatment; months = ibrutinib treatment time. (**B**) Normalized read counts of ATAC-seq at the putative APOBEC3 enhancers are shown in panel (A). N=4 of CLL patients studied at baseline and then overtime to relapse.

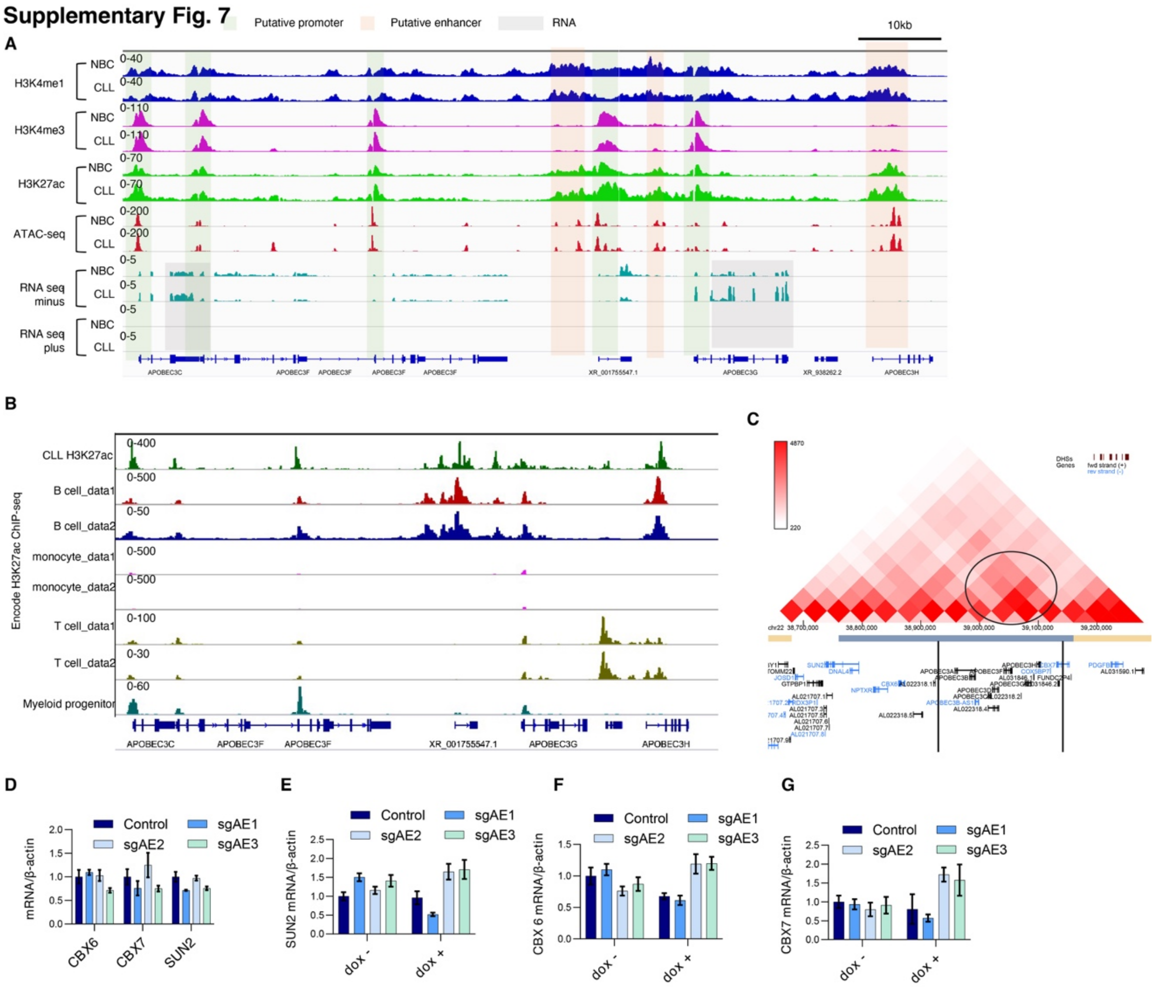

Supplementary Fig. 7 The putative APOBEC3s enhancer signatures are B cell specific.

(**A**) Genome tracks showing H3K4me1, H3K4me3, H3K27ac profiles and ATAC-seq and RNA-seq of putative APOBEC3 enhancers in normal and CLL B cells. Data were downloaded from published dataset EGAD00001004046[5]. (**B**) Genome tracks showing H3K27ac in indicated cells. ChIP-seq data were downloaded from ENCODE project[6]. B cell: ENCBS836CYW, ENCBS400ARI; Monocyte: ENCSR821CPA, ENCSR191YDG; T cell: ENCSR200SSJ, ENCSR245HFY; Myeloid: ENCSR620AZM. (**C**) The Hi-C analysis from GM12878 cells is analyzed and taken from the 3D genome browser site[7]. The boundaries of the topologically associated domains (TADs) and the location of APOBEC3s is indicated below the heatmap. (**D**) RT-qPCR analysis of expression of indicated genes in the AE deleted MEC1 cells. n = 3 independent experiments. (**E-G**) RT-qPCR analysis of expression of indicated genes after inhibition of individual AEs by CRISPRi. n = 3 independent experiments. sgGFP was used as control in panels D-G.

Supplementary Fig. 8

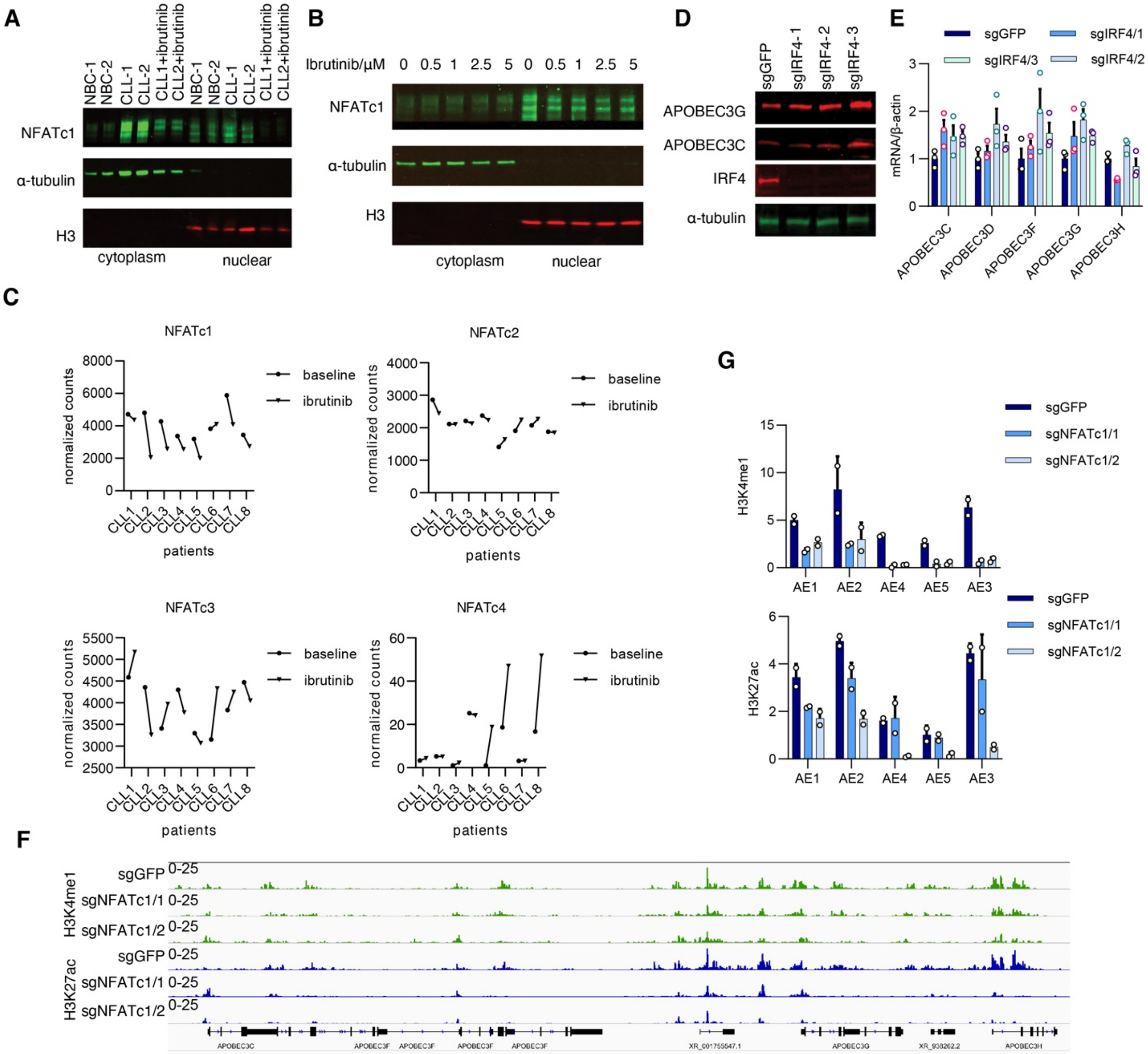

Supplementary Fig. 8 NFATc1 controls the APOBEC3 enhancers activity. (**A**) Western blot showing the NFATc1 level in nuclear and cytoplasm of CLL B cells from patients prior to ibrutinib treatment and then from patients after one-year of continuous ibrutinib treatment. and B cells from normal donors (NBC). (**B**) Western blot showing the NFATc1 level in nuclear and cytoplasm of CLL B cells treated with ibrutinib as indicated for 24 hours. (**C**) NFATc1-4 expression defined by RNA-seq in CLL B cells from patients before (baseline) and after one-year of ibrutinib treatment (ibrutinib). (**D**) Western blot analysis of APOBEC3 expression in IRF4 depleted MEC1 cells. MEC1 cells were infected with sgRNAs targeting IRF4 for 5 days and whole cell lysates were harvested for western blot analysis. (**E**) RT-PCR analysis of APOBEC3 expression in IRF4 depleted MEC1 cells as in panel (D). (**F**) Genome tracks showing H3K4me1

and H3K27ac profiles at APOBEC3 enhancers in NFATc1 depleted JEKO-1 cells. JEKO-1 cells were infected with NFATc1 sgRNAs for 5 days before the cells were harvested for CUT&Tag assay. (**G**) Quantification of the normalized reads of CUT&Tag at the putative APOBEC3 enhancers of panel (F).
